# Translation, cross-cultural adaptation, and measurement properties of the Nepali version of the central sensitization inventory (CSI)

**DOI:** 10.1186/s12883-020-01867-1

**Published:** 2020-07-27

**Authors:** Saurab Sharma, Jyoti Jha, Anupa Pathak, Randy Neblett

**Affiliations:** 1 Department of Physiotherapy, Kathmandu University of School of Medical Sciences, Dhulikhel, Nepal; 2 Centre for Musculoskeletal Outcomes Research, Otago Medical School, University of Otago, Dunedin, New Zealand; 3 Department of Physiotherapy, Grande International Hospital, Kathmandu, Nepal; 4 PRIDE Research Foundation, Dallas, TX USA

**Keywords:** Pain, Musculoskeletal pain, Central nervous system sensitization, Cross-cultural comparison, Pain measurement, Nepal, Developing countries, Clinimetrics, Psychometric properties

## Abstract

**Background:**

Central sensitization is thought to be an important contributing factor in many chronic pain disorders. The Central Sensitization Inventory (CSI) is a patient-reported measure frequently used to assess symptoms related to central sensitization. The aims of the study were to translate and cross-culturally adapt the CSI into Nepali (CSI-NP) and assess its measurement properties.

**Methods:**

The CSI was translated into Nepali using recommended guidelines. The CSI-NP was then administered on 100 Nepalese adults with sub-acute and chronic musculoskeletal pain with additional demographic and pain-related questions. The CSI-Nepali was administered again about 2 weeks later. Four measurement properties of the CSI-NP were evaluated: (1) internal consistency using Cronbach’s alpha, (2) test-retest reliability using intraclass correlation coefficient (ICC_2,1_), (3) measurement errors, and (4) construct validity testing five a priori hypotheses. Confirmation of construct validity was determined if a minimum of 75% of the hypotheses were met.

**Results:**

The CSI was successfully translated into Nepali. Internal consistency and test-retest reliability were both excellent (Cronbach’s alpha = 0.91, and ICC = 0.98). The standard error of measurement was 0.31 and the smallest detectable change was 0.86. Four out of five (80%) a priori hypotheses were met, confirming the construct validity: the CSI-NP correlated strongly with the Pain Catastrophizing Scale total scores (*r* = 0.50); moderately with the total number of pain descriptors (*r* = 0.35); weakly with the Numerical Rating Scale (*r* = 0.25); and women had significantly higher CSI scores than men. However, the CSI scores did not correlate significantly with the total duration of pain, as hypothesized (*r* = 0.10).

**Conclusions:**

The Nepali translation of the CSI demonstrated excellent reliability and construct validity in adults with musculoskeletal pain. It is now available to Nepali health care providers to help assess central sensitization-related signs and symptoms in individuals with musculoskeletal pain in research or clinical practice to advance the understanding of central sensitization in Nepalese samples.

## Background

Musculoskeletal pain is a highly prevalent condition. It is estimated that at least one in three persons experiences it [1]. It is one of the top reasons for years lived with disabilities in both developed and developing countries [2]. A musculoskeletal pain diagnosis increases the risk of mental health problems, other chronic illnesses, and all-cause mortality [3]. Being common in all age groups, including the working age groups, it possesses significant financial costs to both individuals and society [1]. Musculoskeletal pain is the leading cause of disability in Nepal [4] and is the number one reason for hospital admission [5]. Central sensitization (CS) is an important factor that is believed to contribute to many pain disorders, including musculoskeletal pain [6, 7].

CS involves the amplification of pain, and hypersensitivity to other environmental stimuli, within the central nervous system [8]. The Central Sensitization Inventory (CSI) is a relatively new patient-reported outcome measure used to assess somatic and emotional health-related symptoms that have been found to be associated with central sensitization [9]. A cut off score of 40 (out of a total possible score of 100) is often used to screen for the possible presence of central sensitization, so that diagnostic evaluation can be performed, and appropriate treatment can be initiated [10, 11]. The original English version of the CSI, and translated versions in multiple other languages, have demonstrated good to excellent psychometric properties [9, 12–16]. The CSI total scores have been found to be associated with pain catastrophizing, pain intensity, pain interference, depression, anxiety, and quality of life [12, 17–19].

Pain is influenced by culture [20–22]. Therefore, translation, cross-cultural adaptation, and validation of the CSI in Nepali may benefit health care providers who provide assessment and treatment of Nepalese patient populations [23]. Furthermore, the availability of the CSI in Nepali would allow a new dimension of CS-related research in Nepal, which in turn, can contribute to understanding about CS from a population where CS has not been studied [24]. This could then be used in locally adapted pain education programs [25] using a previously proposed guide to improve patient outcomes in individuals with musculoskeletal pain [26].

Therefore, we aimed to translate and culturally adapt the CSI into Nepali (CSI-NP), using recommended guidelines [27], and to further evaluate its measurement properties, including internal consistency, test-retest reliability and construct validity (using hypothesis testing) with four CS-related clinical variables, including pain catastrophizing, pain intensity, duration of pain, and patient-reported pain descriptors. We hypothesized that the CSI-NP would demonstrate good to excellent internal consistency [9, 12, 13, 15, 18], and excellent test-retest reliability [9, 12–15, 18]. We also hypothesized that CSI-NP would positively correlate with pain catastrophizing, pain intensity, and duration of pain [12, 17, 18]. In addition, because female gender has been found to be associated with CS-related disorders, we expected that women would have significantly higher CSI-NP scores compared to men [28–30]. Finally, we explored the association of CSI-NP scores with different types of pain descriptors (e.g., burning, aching, tingling), which, to our knowledge, has not been previously explored.

## Methods

### Study design, and setting

This research was conducted in two steps. In the first step, we performed a translation and cross-cultural adaptation of the CSI into Nepali. In the second step, we assessed the measurement properties of the Nepali version of the CSI. A longitudinal observational study design was used to evaluate the measurement properties of the CSI-NP. Data were collected from two sources: (1) the outpatient Physiotherapy Department of Dhulikhel Hospital, a tertiary care Hospital in Dhulikhel, Nepal, which is 30 km from Kathmandu, and (2) from the community of Bhaktapur, Kathmandu, Lalitpur, and Kavre districts from July to October in 2016.

A consecutive sampling method was used at the hospital, so that every patient presenting with musculoskeletal pain was invited to participate in the study, and those who met the inclusion criteria and consented to participate were included. A purposive sampling method was used to collect data from participants in the community. Participants were recruited door-to-door, and data were collected in their homes from the community sample. The protocol for this study was approved by the Institutional Review Committee of Kathmandu University School of Medical Sciences (ref number 76/16) before data collection began. Informed consent was obtained from every individual before administration of questionnaires. This study was a part of Bachelor of Physiotherapy degree thesis of the second author of this study. All data, including responses to CSI items, were collected in interview format to account for the high rate of illiteracy in Nepal [17].

### Participants

A sample size of more than 50 participants is usually considered adequate for assessing measurement properties of a patient-reported outcome measure [31, 32]. Our goal was to recruit 100 participants for the assessment of the measurement properties in the study. The COSMIN (COnsensus-based Standards for the selection of health status Measurement INstruments) guidelines consider 100 participants “good” for the assessment of internal consistency, test-retest reliability, measurement errors, and construct validity [31].

For the evaluation of measurement properties portion of the study, a total of 115 individuals with self-reported and/or physician-diagnosed musculoskeletal pain were screened for eligibility. Eligible subjects were (1) able to understand and speak Nepali; (2) at least 18 years old; (3) having musculoskeletal pain for at least 1 month (sub-acute or chronic musculoskeletal pain); and (4) having a self-reported pain intensity of at least 3 out of 10 on a 11-point numeric pain rating scale for a minimum of 4 days in the past week [33]. Participants were excluded if they (1) had any recent history of trauma or fracture (within 6 weeks of data collection), (2) were diagnosed as having an acute illness (such as infection), malignancy, or diseases of the central nervous system, cardio-respiratory, gastrointestinal or urogenital system. Of the total sample of 115 subjects who were invited, five declined to participate and 10 were excluded because of the presence of acute pain associated with a neurological or cardiovascular disease, leaving 100 subjects for data analysis.

In addition, a sample of 20 individuals with sub-acute or chronic musculoskeletal pain was recruited from the outpatient Physiotherapy Department of Dhulikhel Hospital, Nepal for the pretesting and cognitive debriefing portion of this study. All study participants consented to participate. No participants were excluded based on low literacy skills.

### Step1: translation of the CSI into Nepali

#### Initial translation process

The original 25 items of the English version of the CSI Part A were translated into Nepali using recommended guidelines [27] using a similar approach to other Nepali translations of patient-reported outcome measures [34, 35]. First, two native Nepali translators (one with a medical background and one without), who were both fluent in English, translated the English version of the CSI into Nepali independently to produce two Nepali translations, T1 and T2. The two translations were synthesized into a single forward translation version (T3) which was facilitated by the first two authors. A written record of the synthesis, process with changes, and decisions was carefully documented. Some words, used to describe medical terms (example, pelvis area, jaw pain, sensitive towards bright light), were either unavailable in Nepali as single words or phrases, or would not be understood by most Nepalese, so a number of experts were consulted, including dentists, gynaecologists, and opthalmologists. Two of the original developers of the CSI were also contacted to clarify the meaning of some of these words in the original English version of the CSI. Finally, the research team pooled all of this information into a common single synthesized version of the CSI, which was used for the back translation into English.

The translation of the Part B of the CSI was deemed “untranslatable” during the preliminary “translatability assessment” of the scale. Translatability assessment is an important pre-requisite of translation and cross-cultural adaptation of outcome measures [36, 37]. This is because Part B of the CSI has names of medical diagnoses that have no comparable Nepali words or translations, are less commonly studied [24] or used in clinical practice in Nepal, and are likely not recognizable by most Nepali-speaking patients. Therefore, a single forward translation of the Part B was performed and it was not subjected to later phases of translations including back-translation or expert committee meeting discussions. All English names of the medical diagnoses were re-written in Devanagari script (i.e. Nepali script).

#### Back translation into English

A translator without a medical background, who was blind to the original English version of the CSI, translated the synthesized Nepali translation (T4) back into English.

#### Expert committee meeting and review

An expert committee was comprised of the researchers and the translators involved in the translation processes. All translation versions were discussed in this meeting, and after the consensus from the committee members, a pre-final Nepali version of the CSI (T5) was created with minimal modification on the choices of words and sentence structure on the T3 version. The English back-translation of this version was then sent to two of the original CSI developers (author RN and Prof Robert Gatchel) for review of the items. Minor changes were made in some items after suggestions from the developers, resulting in a T6 version, which was used for pretesting.

#### Pretesting and cognitive debriefing

The Nepali version of the CSI was tested on 20 individuals with sub-acute or chronic musculoskeletal pain, representing different age categories, both sexes, and a variety of education levels. Guidelines recommend from five to 30 participants adequate for pre-testing and cognitive debriefing [27, 38]. We retrieved no new information after testing 20 participants during the in-depth cognitive interviews. Participants were requested to complete the questionnaire by themselves if they could read and write. The second author (JJ) administered the questionnaire in an interview format for participants who had difficulty reading and writing. All participants were asked to clarify the meaning of the instructions, items, and response options, to assure comprehensibility of the questionnaire for content validity of the CSI [39, 40]. Semantic equivalence was assured in this final version. Minor changes in sentence structure and grammar were made after pretesting and cognitive debriefing, resulting in a final version of the CSI-Nepali (CSI-NP), which can be found in the online only Supplement 1. The original English version of the CSI is available as online Supplement 2.

### Step 2: assessment of measurement properties

After the translation and pre-testing processes were completed, data were collected for assessment of the measurement properties of the CSI-NP. We followed the methodological quality, proposed by the COSMIN recommendations [41]. To account for the low literacy rates in Nepal, all demographic and clinical data were collected by physiotherapists or physiotherapy students in an interview format [42]. The CSI items were also read aloud to each participant, and their answers were recorded by the interviewer. The CSI-NP was re-administered at an interval of approximately 2 weeks for the assessment of test-retest reliability. All 100 participants completed the retest measurement. During the follow-up assessment, participants from the community were interviewed face-to-face, and those recruited from the hospital were interviewed via phone calls.

### Measures used

#### Demographic characteristics

Information regarding participants’ age, gender, marital status, religion, ethnicity, education and occupation were collected to describe the participant characteristics.

#### Assessment of pain

Duration of pain was recorded as the number of weeks since the onset of current pain. Pain intensity was assessed using the Nepali version of the 11-point Numerical Rating Scale (NRS), which has been shown to be valid and reliable, with excellent test-retest reliability (Intraclass correlation coefficient, ICC = 0.81) [43]. Three measures of pain intensity were assessed, including current pain, best pain, and worst pain in the last 24 h [43]. We averaged the three scores into one pain intensity variable, as was done previously in the validation paper of the Nepali version of the NRS [43]. The NRS scores range from “0” = “No pain” to “10” = “Extreme pain”, where a greater score indicates more intense pain.

Quality of pain was assessed with a list of descriptors, which we previously identified as the most common pain quality descriptors in Nepal [44]. These descriptors are similar to the revised short form of the McGill Pain Questionnaire [45]. Specific descriptors included “burning, tingling, piercing, heavy, numb, cramping, stretching, aching, and infection-like.” Participants reported “present” or “absent” on each descriptor. Frequency of their occurrence was also reported. In addition, participants were given the opportunity to provide additional words to describe their pain.

Sites of pain were assessed using a pain diagram. Participants were asked to mark their painful sites on a body chart. The sites of pain were categorized as pain in the neck, shoulder and arm, elbow and forearm, wrist and hand, upper back, lower back, hip and thighs, knee and leg, and ankle and foot.

#### Pain Catastrophizing scale

Pain catastrophizing was assessed using the Nepali version of 13-item Pain Catastrophizing Scale (PCS) [33]. Each item is scored on a Likert scale with “0” = “Never” and “4” = “Always.” The total score ranges from 0 to 52, with higher scores indicating more pain catastrophizing. The Nepali version of the PCS is a valid and reliable measure with good to excellent internal consistency (Cronbach’s alpha = 0.83–0.93) and excellent test-retest reliability (ICC = 0.88–0.90) [33].

#### Central sensitization inventory

Part A of the CSI [9] includes 25-items on a Likert scale, scored from “0” = “Never” to “4” = “Always,” with a total score range of 0 to 100. Higher scores indicate higher central sensitization related symptoms. Five severity level score ranges are available to aid with clinical interpretation (subclinical = 0 to 29; mild = 30 to 39; moderate = 40 to 49; severe = 50 to 59; and extreme = 60 to 100) [46]. Part B, which is not scored, asks if one has been previously diagnosed with a list of common central sensitization-related diagnoses. Part B was not used in the phase 2 of this study.

### Statistical analyses

The raw data were entered into SPSS 24.0 for analyses, and descriptive statistics were computed for demographic and pain variables.

#### Reliability

##### Internal consistency

The internal consistency of the CSI-NP was computed using Cronbach’s alpha. We considered Cronbach’s alpha between 0.80–0.89 to be good, and values equal to 0.90 or more as excellent internal consistency [47].

##### Test-retest reliability

The Intraclass Correlation Coefficient (ICC_2,1_) was used to compute the two-week test-retest reliability using two way mixed model and absolute agreement. The ICC value of more than 0.75 was considered to be excellent test-retest reliability [47].

#### Measurement error

##### Standard error of measurement (SEM)

The SEM was calculated initially by using the formula, SEM = SD * √(1-ICC), where SD = standard deviation of change of CSI-NP from baseline to follow-up.

##### Smallest detectable change (SDC)

The SDC for 95% confidence interval was computed using the formula SEM × Z × √2; where Z is the Z value for the 95% CI and √2 is used to account for the variance of two measurements [48].

##### Bland-Altman plot

The Bland-Altman Plot was created to visualize systematic errors in the baseline and final time-point scores of the CSI [49].

#### Construct validity

##### Hypothesis testing

As indicated earlier, a total of five hypotheses were tested to evaluate the construct validity of the CSI-NP, using a standard hypothesis testing methodology [31, 50]. Spearman correlation coefficient was used to assess the association of the CSI-NP with the PCS, the NRS, duration of pain, and the total number of pain descriptors. We considered *r* values of less than 0.30 as a weak correlation, 0.30–0.49 as a moderate correlation and values of 0.50 or higher as a strong correlation [51]. An unpaired *t-test* was used to assess if women had significantly higher CSI score than men. The results are reported as “hypothesis confirmed” or “hypothesis not confirmed”. The total number of met hypotheses were added and reported as percentages. If the total percentage was more than 75%, we confirmed the construct validity of the CSI-NP as per the COSMIN recommendations [32].

## Results

### Translation and cross-cultural adaptation

Three items required cross-cultural adaptation during the translation phase, with input from two of the developers of the CSI. Item 19, “I have pain in my jaw,” was difficult to translate because a single Nepali word for “jaw” does not exist. After discussion within the translation team and a dentist, the item was translated as “I have pain in the muscles and joints around my molar teeth.” Item 25, “I have pain in my pelvic area,” was translated as “I have pain between my hips (near my private body parts)” because there was no agreement on a Nepali word for pelvic area that is easily understandable. Finally, the Nepali translation of item 24, “I suffered trauma as a child,” only reflected physical trauma in the initial Nepali translation. Therefore, to assure the conceptual equivalence we explicitly added “physical or psychological” before “trauma” to clarify that trauma could be either physical or psychological. Translation of the part B of the CSI was difficult because Nepali words for most of the medical conditions were not available in Nepali, therefore the vigorous forward and backward translation of part B of the CSI was not performed.

### Demographic and pain characteristics

The mean age of the study participants was 42.01 (14.61 SD) years. The majority of participants were female (67%), married (76%), and Hindu by religion (79%). Almost half of the participants were unemployed (47%). The mean duration of pain was 111 weeks, the median was 24 weeks, and the range was 4 to 1280 weeks. The most common sites of pain were the knee (32%) followed by the low back (24%). Additional socio-demographic details are presented in Table 1.

**Table 1:** Socio-demographic characteristics with distribution of site of pain

Variables	Frequency N (%)
**Sex**	
Men	33 (33%)
Women	67 (67%)
**Marital status**	
Married	76 (76%)
Single	16 (16%)
Separated	1 (1%)
Missing	7 (7%)
**Religion**	
Hindu	79 (79%)
Buddhist	11 (11%)
Others	3 (3%)
Missing	7 (7%)
**Ethnicity**	
Brahmin	31 (31%)
Newar	28 (28%)
Chettri	18 (18%)
Tamang	9 (9%)
Others	7 (7%)
Missing	7 (7%)
**Occupation**	
Unemployed	47 (47%)
Office	20 (20%)
Agriculture	11 (11%)
Student	8 (8%)
Others	7 (7%)
Missing data	7 (7%)
**Education**	
Never went to school	30 (30%)
Less than 5 years	15 (15%)
6–12 years	20 (20%)
Bachelor and above	28 (28%)
Missing data	7 (7%)
**Site of pain**	
Knee and leg	32 (32%)
Low back	24 (24%)
Two or more pain sites	16 (16%)
Upper back	7 (7%)
Ankle and Foot	6 (6%)
Shoulder	5 (5%)
Neck	4 (4%)
Wrist	4 (4%)
Hip and thigh	2 (2%)

Total mean score for the CSI-NP was 22.85 (SD 12.35) with scores ranging from 0 to 59. A majority of the sample (75%) scored in the subclinical severity range (scores < 29). Only 14% were categorized as mild, 8% as moderate, and 3% as severe central sensitization related symptoms. No subjects scored in the extreme category. The total mean score for the PCS was 18.51 (SD 11.84; range 0–52) and the NRS was 5.17 (SD 1.85, range 2–10).

### Reliability

Internal consistency of the CSI-NP was good (Cronbach’s alpha = 0.87). Test-retest reliability of the CSI-NP was excellent with ICC = 0.98 (95% CI: 0.97, 0.99).

### Measurement error

**Fig. 1:**
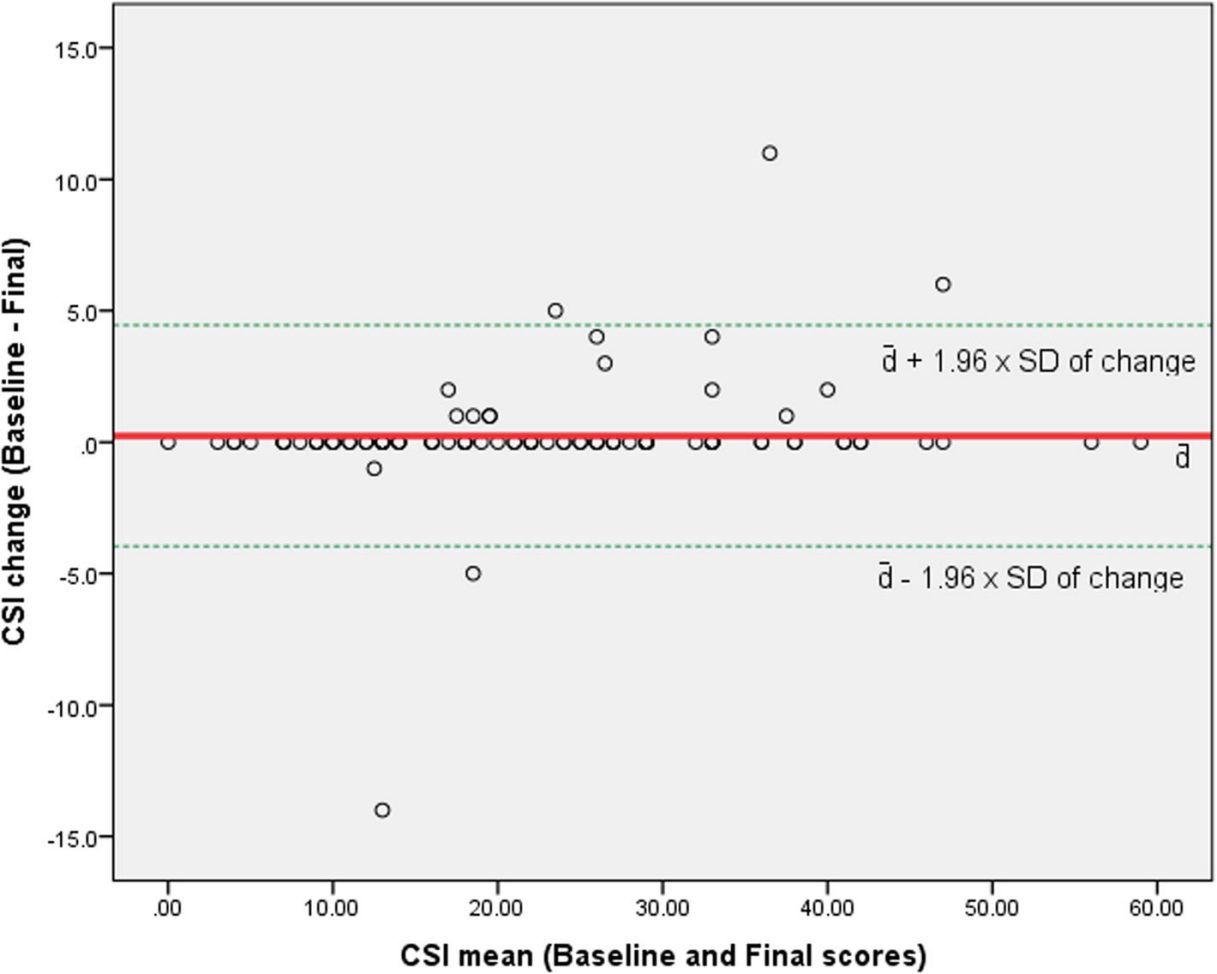
Bland-Altman Plot for the Nepali Central Sensitization Inventory. Note: Y-axis is the change of the CSI scores and X-axis is the mean of the CSI scores at the baseline and final measurements. Solid red line is the mean change of score (d̄); and dotted green lines are d̄ ± Z x SD_change_ (where Z = 1.96 for 95% confidence interval)

Standard error of measurement was 0.31. Smallest detectable change (SDC) was 0.86. The Bland-Altman Plot, shown in Fig. 1**,** presents the distribution and variability in the CSI scores in the baseline and final measurements for each study participant.

### Construct validity

As shown in Table 2, a total of 4 of 5 a priori hypotheses (80%) were met, confirming the construct validity of the CSI-NP. The CSI-NP correlated strongly with PCS with (*r* = 0.50, *P* < 0.001), moderately with the total number of pain descriptors (*r* = 0.35, *P* < 0.001), and weakly with NRS pain intensity (*r* = 0.25, *P* = 0.013). Mean CSI-NP scores were significantly higher in women (25.21; SD 12.23) compared to the men (18.06; SD 10.87). The correlation of the CSI-NP with pain duration (*r* = 0.10; *P* = 0.315) was weak and non-significant.

**Table 2:** Results of hypotheses testing for construct validity of the CSI-NP (*N* = 100)

Scale	Construct validity (correlation with baseline scores)		
	Hypothesis	Results	Hypothesis confirmed?
**CSI-NP**	Positive weak to strong associations with pain intensity	*r* = 0.25 (P = 0.013)	Yes
	Positive weak to strong associations with pain catastrophizing	*r* = 0.50 (P < 0.001)	Yes
	Positive weak to moderate associations with duration of pain	*r* = 0.10 (P = 0.315)	No
	Positive weak to moderate associations with total number of types of pain	*r* = 0.35 (*P* < 0.001)	Yes
	Women would have significantly higher CSI scores than men	Women had significantly higher CSI scores than men [Mean difference = 7.15 (95% CI: 2.11, 12.19); *P* = 0.005]	Yes
	**Total hypothesis met**		**4 of 5 (80%)**

*Abbreviations*: *CS* Central Sensitization, *CSI-NP* Nepali version of Central Sensitization Inventory

*r* = < 0.30 weak, 0.30–0.49 moderate, ≥0.50 strong correlations

Exploratory analysis revealed that CSI-NP scores were frequently and significantly associated with three pain descriptors, including heavy, tingling, and infection-like pain (throbbing pain), but not with other types of pain, including achy, piercing, stretching, numb, cramping, and burning (see Table 3).

**Table 3:** Pain quality and their correlation with the CSI-NP (N = 100)

Pain quality	N	***r*** with CSI-NP	***P***
*Kat-kat* (Achy)	64	0.20	0.052
Piercing pain	57	0.05	0.587
**Heavy pain**	52	**0.22**^**a**^	**0.032**
Stretching pain	40	0.11	0.300
**Tingling**	39	**0.22**^**a**^	**0.027**
Numb	37	0.19	0.065
Cramping pain	30	0.19	0.063
***Infection-like pain*** **(throbbing)**	28	**0.22**^**a**^	**0.026**
Burning	26	0.16	0.103

*Abbreviations.* N, total number of participants reporting “Yes” on the specific quality of pain; r, correlation coefficient

^a^Indicates significance level at < 0.05. Significant associations with the CSI-NP are highlighted in bold text

## Discussion

The study aimed to translate and cross-culturally adapt the CSI into Nepali and evaluate its measurement properties. The Nepali translation of the CSI was shown to be a reliable and a valid measure with small measurement errors. In general, the CSI scores for the Nepalese sample were lower compared to samples collected from other geographic regions and other pain populations [9, 13]. In fact, the mean CSI-NP score in our clinical sample (22.85) was smaller than healthy control samples from the USA (28.90) [9] and Brazil (37.14) [12]. Mean CSI scores have varied in other clinical samples (with individuals with fibromyalgia generally scoring the highest and those with localized pain scoring lower) and with different cultural regions (with generally higher scores in Western countries and generally lower scores in Eastern countries) [19]. For instance, a Japanese chronic pain sample scored in a similar range as our clinical sample (21.91) [18]. Perhaps there are cultural differences in the perception and reporting of central sensitization symptoms. Future studies may want to concurrently collect data on CSI between two or more countries or cultures using the identical study designs in order to determine the true differences in the CSI scores across countries or cultures as we previously recommended [52].

### Reliability

We found excellent reliability (internal consistency and test-retest reliability of the CSI-NP consistent with multiple language versions of the CSI [9, 12–15, 18, 53]. The internal consistency of the CSI-NP was comparable to other language versions, whereas, the test-retest reliability was larger than for other language versions including the Brazilian Portuguese, Dutch, English, French, Serbian, and Spanish (ICC = 0.82 to 0.95) [9, 12–15, 53] but comparable to Greek and Gujrati versions [54, 55]. The primary reason for a higher test-retest reliability may be that the researcher who administered the follow-up CSI measure provided the values of the baseline measurement to each participant. This could have confounded the follow-up scores, yielding high levels reliability, as reflected in the Bland-Altman Plot in Fig. 1. Also, unlike other CSI studies, the CSI in the present study was administered in an interview format. Future studies could explore if there are differences in test-retest reliability of the CSI-NP in a self-reported format. We were unable to analyze reliability separately for face-to-face and telephone interviews. In a previous and similar validation study, we found no differences in test-retest reliability of the Nepali Disability of Arm, Shoulder, and Hand Questionnaire [56]. Further studies may assess the reliability separately for face-to-face administration of CSI-NP.

### Measurement error

Measurement errors, although important measurement property of a patient-reported outcome measure, is rarely measured in the CSI studies. The CSI-NP showed standard error of measurement (SEM = 0.86) smaller than other language versions (SEM = 1.84 to 3.16) [13, 14, 54, 55]. The SDC (= 8.86) in the current study was also smaller than two previous studies (SDC = 5.90 and 7.83) [13, 55]. It could be because of the same reasons that accounted for high test-retest reliability as described above. Based on the current findings, any change score more than 1 unit (out of 100) may be viewed as true change for the Nepali version of the CSI. Future studies should explore measurement errors for other language versions of the CSI and compare against minimum important change scores, which should ideally exceed the SDC values [31, 57]. The assessment of measurement error should be repeated in other Nepalese samples without disclosing the baseline scores to the patients during the retest assessment, which will potentially provide more accurate estimate of measurement errors.

### Construct validity

Eighty percentage (four of five) of a priori hypotheses were met, which support the construct validity of the CSI-NP based on criteria proposed by Terwee and colleagues [32]. Construct validity of the CSI has primarily been assessed using factor analyses in previous studies. The form of hypothesis testing used in the present study is another recommended approach [31, 50] for assessing the construct validity of a patient-reported outcome measures.

The hypotheses were made based on the direction and magnitude of associations. Due to lesser number of similar associations being tested in previous research, the magnitude of association hypothesized were open wider. Although the hypotheses on the association of the CSI-NP with pain intensity, pain catastrophizing were met as hypothesized, the strength of correlations we found are varied. For example, the correlation of the CSI-NP with pain intensity (r = 0.25) was lower than the Dutch and Japanese versions (*r* = 0.51 and 0.42 respectively) [15, 18]. Similarly, correlation of the CSI-NP with pain catastrophizing (*r* = 0.50) was similar to the Dutch version (*r* = 0.52) but lesser than Brazilian Portuguese version (*r* = 0.68). Our hypothesis that women experience more CS related symptoms is consistent with previous literature [28–30]. The a priori hypothesis that the CSI scores would positively be associated with total number of types of pain was met, which provides an indication that individuals with higher CSI scores are more likely to report more number of types of pain (e.g., achy, heavy, tingling, throbbing). If this finding is consistently replicated, it could stand as an important feature of CS that can easily be identified during communication with patients. Although CS is expected to be associated to chronicity, we did not find a significant association between the CSI-NP and total duration of pain. The association of the CSI with the duration of pain and total number of types of pain need further exploration.

Exploratory analyses for the association of CSI scores with heavy pain, tingling pain, and throbbing pain is interesting, and should be confirmed in further studies to assess if particular types of pain are associated more strongly with central sensitization signs and symptoms than the other types of pain. If this is the case, type of pain then could help flag central sensitization related signs and symptoms.

### Strengths and limitations

Despite the methodological strengths, i.e. adoption of standard recommended guidelines to translate and cross-culturally adapt CSI into Nepali [27], and compliance with COSMIN recommendations [31, 41] to perform analyses for measurement properties, the limitations of the study should be carefully considered. First, we did not perform a factor analysis of the CSI-NP in the current study because 150 additional participants would have been required based on recommended guidelines (with 10 participants per item [31]). Because of the feasibility reasons, it was not practical to collect an additional 150 subjects simply for the purpose of a factor analysis. Second, we did not perform a responsiveness analysis and the minimum important change scores for the CSI-NP. Future studies might consider the assessment of responsiveness to change and minimum important change scores to be able to better interpret the change of the CSI-NP scores after interventions to target central sensitization in the individuals with musculoskeletal pain. Third, we did not assure that the patients were unchanged between the test and re-test interval for the assessment of test-retest reliability and measurement errors, as recommended by the COSMIN guidelines [31, 41]. It was our assumption that a duration of 2 weeks was not enough time for any significant change to occur in the construct of central sensitization in all or most of the study participants. Fourth, the CSI-NP was tested only on adults with musculoskeletal pain, therefore, the questionnaire should not be assumed valid for other populations (e.g., pediatric patients and patients with cancer-related pain). Fifth, we did not administer the CSI on normal healthy individuals to explore if the CSI scores discriminated individuals with and without pain and if the scores would differ from individuals with chronic pain syndromes. Future studies should consider the assessment of the CSI scores in normal healthy individuals to see how the CSI scores differ between samples with or without pain. Finally, because of non-random sampling methods used to recruit participants from the community, two thirds of the study participants recruited in the current sample were women. The readers should be wary of this when considering the generalizability of the study findings. The future studies may consider random sampling approaches to recruit study participants from community settings.

## Conclusions

The CSI was successfully translated into Nepali for the first time. The CSI scores were shown to be reliable and valid and that it can be used to assess signs and symptoms related to central sensitization in adults with musculoskeletal pain in Nepal in clinical practice or research. Future studies on the CSI-NP should assess treatment responsiveness, compute a minimum important change score, compare if that score is larger than the values of measurement errors, and validate the CSI in other pain populations to extend its clinical and research use.

## Supplementary information

**Additional file 1.** Nepali-CSI final version.

**Additional file 2.** CSI English version.

## Data Availability

The dataset used and/or analysed during the current study is available from the corresponding author on reasonable request.

## Abbreviations

CI: Confidence Interval
COSMIN: COnsensus-based Standards for the selection of health status Measurement INstruments
CS: Central Sensitization
CSI: Central Sensitization Inventory
CSI-NP: The Nepali version of the Central Sensitization Inventory
ICC: Intraclass Correlation Coefficient
NRS: Numerical Rating Scale
PCS: Pain Catastrophizing Scale
SD: Standard Deviation
SDC: Smallest Detectable Change
SEM: Standard Error of Measurement
SPSS: Statistical Package for Social Sciences
