# Supplementary material for: Translation, cross-cultural adaptation, and measurement properties of the Nepali version of the central sensitization inventory (CSI)
Source: BMC Neurol. 2020 Jul 27;20:286. doi: 10.1186/s12883-020-01867-1 (PMC7385946; doi:10.1186/s12883-020-01867-1)
Supplement: Supplementary file 1 — Additional file 1. Nepali-CSI final version. [file 12883_2020_1867_MOESM1_ESM.pdf]

सेन्ट्रल सेन्सीटाइजेसन इनभेनटोरी (भाग - क)

कृपया हरेक वाक्यको दायाँतिर रहेको उचित उत्तरमा (√)चिन्ह लगाउनुहोस् ।

	कहिले पनि हुन्न	बिरलै	कहिले काहिँ	धेरै जसो	सधैँ
१. जब म सुतेर उठ्छु, म थकित र ताजगी नभएको महशुस गर्दछु ।					
२. म मेरो मांसपेशी कक्रक्क परेको र दुखेको महशुस गर्छु ।					
३. मलाई तनाबको दौरा पर्छ ।					
४. म आफ्नो दारा किट्छु अथवा कस्छु ।					
५. मलाई पखाला र / वा कब्जियतको समस्या छ ।					
६. मलाई मेरो दैनिक कार्य गर्न मद्दत चाहिन्छ ।					
७. तेजिलो प्रकाशप्रति म संबेदनशील छु ।					
८. जब म शारीरिक रुपले सक्रिय हुन्छु, म धेरै सजिलै थाक्छु ।					
९. म मेरो पुरै शरीरमा दुखाई महशुस गर्छु ।					
१०. मलाई टाउको दुख्ने समस्याहरु छ ।					
११. जब म पिसाब फेर्छु, मेरो मुत्रथैलीमा असजिलो अथवा पोलेको महशुस गर्छु ।					
१२. म राम्रोसँग सुत्दिनँ ।					
१३. मलाई एकाग्रता (ध्यान) पुर्याउन गाह्रो छ ।					
१४. मलाई छालाको समस्या छ, जस्तै फुस्रोपन, चिलाउने अथवा रातो (डाबर) हुने ।					
१५. तनाबले मेरो शारीरिक लक्षणहरु बढ्छ ।					
१६. म दु:खी वा उदास महशुस गर्छु ।					
१७. मेरो शारीरिक उर्जामा कमि छ ।					
१८. मेरो गर्दन र काँधका मांसपेशीमा कडापन छ ।					
१९. मेरो बंगाराको जोर्नी तथा मांसपेशीमा दुखाई छ ।					
२०. केहि गन्धहरु जस्तै अत्तरले मलाई रिंगटा र वाकवाकि महशुस गराउँछ ।					
२१. मलाई बारम्बार पिसाब फेर्नु पर्छ ।					
२२. जब म राति निदाउन कोशिस गर्छु, मेरो खुट्टाहरुमा असहज र बेचैनी महशुस गर्छु ।					
२३. मलाई कुराहरु सम्झिन गाह्रो हुन्छ ।					
२४. बाल्य अवस्थामा मैले (मानसिक वा शारिरीक) चोट भोगेको छु ।					
२५. मेरो दुई कट्टीको बीचको भागमा (अथवा गुप्ताङ्गमा) दुखाई छ ।					
**जम्मा अंक**					

The Nepali translation of the Central Sensitization Inventory (CSI)

Available from Sharma S, Jha J, Pathak P, Neblett R. Translation, cross-cultural adaptation, and measurement properties of the Nepali version of the Central Sensitization Inventory (CSI). 2020. BMC Neurology. https://doi.org/10.1186/s12883-020-01867-1

सेन्ट्रल सेन्सीटाइजेसन इनभेनटोरी (भाग - ख)

नामः ___________________________________

मितिः ___________________________________

के तपाईलाई तपाईको डाक्टरले तल मध्यको कुनै समस्या रहेको भनेर औंल्याउनु भएको छ? कृपया हरेक स्वास्थ्य समस्याको दायाँतिर रहेको उचित उत्तरमा (√)चिन्ह लगाउनुहोस् र समस्याको निदान भएको वर्ष पनि लेख्नुहोस् ।

	**स्वास्थ्य समस्याहरु**	**छैन**	**छ**	**पत्ता लागेको मिति लेख्नुहोस्**
१	रेस्टलेस लेग सिन्ड्रोम			
२	क्रोनिक फटिग सिन्ड्रोम			
३	फाइब्रोमायाल्जिया			
४	टेम्पोरोम्यान्दिबुलर जोइन्त डिसअर्डर			
५	माइग्रेन वा टेन्सन हेडएक (टाउको दुख्ने समस्या)			
६	इरिटेबल बावल सिन्ड्रम			
७	मल्टिपल केमिकल सेन्सिटिविटी			
८	गर्दनको चोट (जस्तै विप्ल्याश्)			
९	एन्जाइटी वा प्यानिक अट्याक (चिन्ता वा तनावको दैरा)			
१०	डिप्रेसन (उदासिन्ता)			

The Nepali translation of the Central Sensitization Inventory (CSI)

Available from Sharma S, Jha J, Pathak P, Neblett R. Translation, cross-cultural adaptation, and measurement properties of the Nepali version of the Central Sensitization Inventory (CSI). 2020. BMC Neurology. https://doi.org/10.1186/s12883-020-01867-1
