# Supplementary material for: Translation, cross-cultural adaptation, and measurement properties of the Nepali version of the central sensitization inventory (CSI)
Source: BMC Neurol. 2020 Jul 27;20:286. doi: 10.1186/s12883-020-01867-1 (PMC7385946; doi:10.1186/s12883-020-01867-1)
Supplement: Supplementary file 2 — Additional file 2. CSI English version. [file 12883_2020_1867_MOESM2_ESM.pdf]

CENTRAL SENSITIZATION INVENTORY: PART A

Name: ____ Date: _____________________

Please circle the best response to the right of each statement.

1	I feel tired and unrefreshed when I wake from sleeping.	Never	Rarely	Sometimes	Often	Always
2	My muscles feel stiff and achy.	Never	Rarely	Sometimes	Often	Always
3	I have anxiety attacks.	Never	Rarely	Sometimes	Often	Always
4	I grind or clench my teeth.	Never	Rarely	Sometimes	Often	Always
5	I have problems with diarrhea and/or constipation.	Never	Rarely	Sometimes	Often	Always
6	I need help in performing my daily activities.	Never	Rarely	Sometimes	Often	Always
7	I am sensitive to bright lights.	Never	Rarely	Sometimes	Often	Always
8	I get tired very easily when I am physically active.	Never	Rarely	Sometimes	Often	Always
9	I feel pain all over my body.	Never	Rarely	Sometimes	Often	Always
10	I have headaches.	Never	Rarely	Sometimes	Often	Always
11	I feel discomfort in my bladder and/or burning when I urinate.	Never	Rarely	Sometimes	Often	Always
12	I do not sleep well.	Never	Rarely	Sometimes	Often	Always
13	I have difficulty concentrating.	Never	Rarely	Sometimes	Often	Always
14	I have skin problems such as dryness, itchiness, or rashes.	Never	Rarely	Sometimes	Often	Always
15	Stress makes my physical symptoms get worse.	Never	Rarely	Sometimes	Often	Always
16	I feel sad or depressed.	Never	Rarely	Sometimes	Often	Always
17	I have low energy.	Never	Rarely	Sometimes	Often	Always
18	I have muscle tension in my neck and shoulders.	Never	Rarely	Sometimes	Often	Always
19	I have pain in my jaw.	Never	Rarely	Sometimes	Often	Always
20	Certain smells, such as perfumes, make me feel dizzy and nauseated.	Never	Rarely	Sometimes	Often	Always
21	I have to urinate frequently.	Never	Rarely	Sometimes	Often	Always
22	My legs feel uncomfortable and restless when I am trying to go to sleep at night.	Never	Rarely	Sometimes	Often	Always
23	I have difficulty remembering things.	Never	Rarely	Sometimes	Often	Always
24	I suffered trauma as a child.	Never	Rarely	Sometimes	Often	Always
25	I have pain in my pelvic area.	Never	Rarely	Sometimes	Often	Always
					**Total=**	

Rev. 6-3-2015

CENTRAL SENSITIZATION INVENTORY: PART B

Name: ___________________________________

Date: ____________________________________

Have you been diagnosed by a doctor with any of the following disorders?

Please check the box to the right for each diagnosis and write the year of the diagnosis.

		NO	YES	Year Diagnosed
1	Restless Leg Syndrome			
2	Chronic Fatigue Syndrome			
3	Fibromyalgia			
4	Temporomandibular Joint Disorder (TMJ)			
5	Migraine or tension headaches			
6	Irritable Bowel Syndrome			
7	Multiple Chemical Sensitivities			
8	Neck Injury (including whiplash)			
9	Anxiety or Panic Attacks			
10	Depression			
